# Antigen-Specific Treg Therapy in Type 1 Diabetes – Challenges and Opportunities

**DOI:** 10.3389/fimmu.2021.712870

**Published:** 2021-07-22

**Authors:** Isabelle Serr, Felix Drost, Benjamin Schubert, Carolin Daniel

**Affiliations:** ^1^ Group Immune Tolerance in Type 1 Diabetes, Helmholtz Diabetes Center at Helmholtz Zentrum München, Institute of Diabetes Research, Munich, Germany; ^2^ Deutsches Zentrum für Diabetesforschung (DZD), Neuherberg, Germany; ^3^ School of Life Sciences Weihenstephan, Technische Universität München, Garching bei München, Germany; ^4^ Institute of Computational Biology, Helmholtz Zentrum München, German Research Center for Environmental Health, Neuherberg, Germany; ^5^ Department of Mathematics, Technische Universität München, Garching bei München, Germany; ^6^ Division of Clinical Pharmacology, Department of Medicine IV, Ludwig-Maximilians-Universität München, Munich, Germany

**Keywords:** antigen-specific Treg therapy, autoimmunity, T1D, microRNAs, tissue Tregs, single-cell multi-omics integration, TCR specificity prediction

## Abstract

Regulatory T cells (Tregs) are key mediators of peripheral self-tolerance and alterations in their frequencies, stability, and function have been linked to autoimmunity. The antigen-specific induction of Tregs is a long-envisioned goal for the treatment of autoimmune diseases given reduced side effects compared to general immunosuppressive therapies. However, the translation of antigen-specific Treg inducing therapies for the treatment or prevention of autoimmune diseases into the clinic remains challenging. In this mini review, we will discuss promising results for antigen-specific Treg therapies in allergy and specific challenges for such therapies in autoimmune diseases, with a focus on type 1 diabetes (T1D). We will furthermore discuss opportunities for antigen-specific Treg therapies in T1D, including combinatorial strategies and tissue-specific Treg targeting. Specifically, we will highlight recent advances in miRNA-targeting as a means to foster Tregs in autoimmunity. Additionally, we will discuss advances and perspectives of computational strategies for the detailed analysis of tissue-specific Tregs on the single-cell level.

## Introduction

The body’s immune system has evolved to effectively defeat and destroy infiltrating foreign pathogens. In order to prevent autoimmune reactions directed against the body’s own cells, our immune system employs sophisticated mechanisms of self-tolerance. On the T cell level, self-tolerance is executed in the thymus by deletion of T cells with self-reactive TCRs (central tolerance). Outside of the thymus, peripheral tolerance is maintained by specialized cells, including so-called regulatory T cells (Tregs). Tregs are characterized by the high expression of the interleukin-2-receptor-aplpha chain (CD25) and the transcription factor Foxp3, which is the master regulator of Tregs phenotype and function (1–4). The critical importance of Tregs for the maintenance of self-tolerance is illustrated by severe multi-organ autoimmunity in humans with the immune dysregulation, polyendocrinopathy, enteropathy, X-linked syndrome (IPEX) (5) and mice with Scurfy mutations (6), both resulting from mutations in the *Foxp3* gene. Tregs develop in the thymus, referring to thymic Tregs (tTregs), and harbor a TCR repertoire that is skewed towards self-antigens. Additionally, Tregs can likewise be induced in the periphery in an antigen-specific manner, so called peripheral Tregs (pTregs), with a TCR repertoire different from their tTreg counterparts (7). Considerable research has been conducted in order to induce disease-relevant antigen-specific Tregs with the goal to restore mechanisms of tolerance and interfere with unwanted immune reactions in allergies and autoimmunity. Accordingly, we and others have shown that Treg induction requires stimulation *via* the TCR and it has become apparent that fine-tuned TCR signals are needed to efficiently induce Tregs (8–11). Here, we will discuss promising results for antigen-specific Treg therapies in allergy and specific challenges for such therapies in autoimmune diseases, with a focus on type 1 diabetes (T1D) as well as opportunities for antigen-specific Treg therapies in T1D.

## Advances in Antigen-Specific Treg Therapies in Allergy

Antigen-specific therapy is a long-envisioned goal for the treatment or prevention of autoimmune diseases. The ability of Tregs to regulate immune responses not only *via* direct inhibition of effector T cells with the same specificity but also *via* modulation of antigen-presenting cell (APCs), a process called bystander suppression, makes Tregs an important target for tolerizing therapies (12). Currently, approaches based either on the expansion, manipulation and transfer of autologous Tregs as well as the *in vivo* induction with antigen are extensively studied. While the *ex vivo* expansion of polyclonal Tregs has proven to be safe in the clinic the efficacy is largely dependent on disease-relevant antigen-specific Tregs. However, their very low frequency in the case of autoimmune diseases necessitates the manipulation of Tregs before transfer [reviewed in (13)]. This includes the forced expression of FOXP3 in autoantigen-specific effector T cells as well as the expression of disease relevant TCRs on isolated Tregs [reviewed in (13)]. Although results from preclinical studies are promising, the long-term fate of these engineered Tregs is not fully understood and especially the differentiation into pro-inflammatory lineages might be a safety concern. The alternative of induction of Tregs with antigen administered directly to the patients is more cost-effective and its safety has been demonstrated in a variety of clinical trials. Even though clinical translation of such tolerizing therapies has been challenging, several examples relying on different forms of antigen-delivery and tolerization protocols from pre-clinical and clinical trials highlight the potential of such strategies.

Desensitization to allergens is a common practice for the treatment of severe allergies. However, only a few studies have addressed the effect of such antigen-specific desensitization protocols on Tregs. Importantly, oral immunotherapy with peanut proteins in allergic patients led to an increase in peanut protein-specific FOXP3^+^ Tregs within peripheral blood mononuclear cells (PBMCs) 6 and 12 months after the treatment started (14). Interestingly, in a follow-up study focusing more specifically on Tregs, it became evident that the increased frequencies of peanut-protein specific Tregs were associated with enhanced DNA demethylation of the *FOXP3* locus (15), a measure for maintenance of FOXP3 expression and therefore for the stability of the Treg phenotype (16). These findings highlight that antigen-specific therapy can not only enhance Treg frequencies but also positively affect Treg characteristics including their stability.

## Challenges for Antigen-Specific Treg Therapy in Autoimmunity and T1D

Autoimmune diseases like T1D affect millions of people worldwide with a steadily rising incidence. Currently, curative treatments for autoimmune diseases do not exist and available therapies rely on the treatment of symptoms often involving immunosuppressive reagents that can have severe side effects. The antigen-specific induction of disease-relevant Tregs offers the opportunity to restore natural tolerance mechanisms in the absence of immune side effects induced by general immune suppression and is therefore a long-standing goal for the treatment or prevention of autoimmune diseases. We were able to demonstrate that in the peripheral blood of children at risk to develop T1D, insulin-specific Treg frequencies are reduced during the onset of islet autoimmunity, while higher frequencies are associated with a slow progression to clinically overt T1D (17). These findings directly support the concept of inducing these insulin-specific Tregs to delay the progression to clinically symptomatic disease. However, the translation of antigen-specific Treg therapies for autoimmune diseases into the clinic remains challenging and most studies using oral insulin treatments for tolerization in T1D conducted so far failed to meet their primary outcome (18, 19). Nevertheless, post-hoc analysis revealed a delay in progression in a subset of these treated participants (20). One analytical caveat of clinical trials studying Treg therapies has been the divergence of protocols for Treg identification in peripheral blood. While in the mouse setting Foxp3 is expressed exclusively by Tregs, human effector T cells can transiently express intermediate levels of FOXP3. Accordingly, most researchers characterize human Tregs as CD25^+^CD127^low^FOXP3^+^. It has become apparent though, that even those more stringently defined Tregs are heterogeneous in their composition. Not only can Tregs co-express classical effector T cell transcription factors (e.g. TBET, RORC, GATA3) which affects their migration and function, but they also vary in their activation state and functionality. This is especially evident in the divergent expression of CD45RA, with CD45RA^-^ Tregs being antigen-experienced and having a higher suppressive activity [reviewed in (7)]. According to this heterogeneity, divergent markers have been used for the identification of Tregs in clinical trials which contributes to the difficulties in assessing translatability. Importantly, researchers are starting to analyze antigen-specific immune responses in such clinical trials in more mechanistic detail, which will help to define critical parameters, such as the optimal dosing of oral insulin. Additionally, other factors need to be critically considered, including the route of administration and the chosen antigen but also the time point of administration within the disease course.

We know from murine studies that the efficient *de novo* induction of Tregs from naïve T cells *in vivo* requires the stimulation with a strong-agonistic ligand for the TCR supplied under subimmunogenic conditions (8, 9). Higher immunogenic doses of antigen on the other hand activate the Pi3k-Akt-mTOR pathway, thereby directly inhibiting Treg induction (10). We used immunodeficient HLA-DQ8-transgenic NOD-Scid-IL2Rγ knockout (NSG) mice reconstituted with human hematopoietic stem cells to study requirements for human Treg induction *in vivo*. Importantly, these humanized mice develop a functional human immune system, including the positive selection of autoreactive insulin-specific CD4^+^ T cells in the thymus (17, 21). Using this system under steady state conditions in the absence of autoimmune activation, we were able to demonstrate that, similar to the murine setting, subimmunogenic doses of strong-agonistic insulin variants are able to induce human Tregs *in vivo* (17).

In contrast to the steady state, we demonstrated that during the onset of islet autoimmunity the capacity to induce Tregs from naïve T cells from peripheral blood is significantly impaired (22). Importantly, this impairment in Treg induction was not limited to the insulin-specific population, but was likewise observed for hemagglutinin-specific and polyclonal Treg induction, highlighting a broad defect in Treg induction (22). Furthermore, we were able to show that a reduction in the activation threshold of insulin-specific T cells during the onset of islet autoimmunity limits the possibility of subimmunogenic stimulation for efficient Treg induction (22). Apart from defects in Treg induction during islet autoimmunity, we likewise observed reduced Treg stability as indicated by increased DNA methylation of the conserved non-coding sequence 2 (CNS2) of the *Foxp3* locus both in non-obese diabetic mice (NOD, mouse model for T1D) with islet autoimmunity as well as in children with overt T1D (23). The *Foxp3* CNS2 is completely demethylated in stable Tregs, while its methylation leads to the loss of Foxp3 expression and the Treg phenotype (16). Importantly, this defect in Treg stability in NOD mice was observed already at a young age, shortly after weaning, indicating a possible causative role in disease development and progression as opposed to a mere consequence of the ongoing autoimmune process (23). The identified impairments in Treg induction and stability directly highlight the importance of considering the time point of administration of antigen-specific Treg inducing therapies. Our *in vitro* and *ex vivo* data suggest limitations in the efficacy of such treatments during the first years after development of islet autoimmunity. In addition, these findings strengthen the rationale of considering preventive strategies in genetically at-risk patients, before the onset of overt islet autoimmunity, for future antigen-specific Treg targeting in man. Accordingly, for T1D pilot results from the Pre-POINT study, the first study to administer daily oral insulin to children at risk to develop T1D, but before the start of the autoimmune reaction, resulted in enhanced frequencies of insulin-specific CD4^+^ T cells with regulatory features (24). These preliminary results are currently further investigated in the larger POINT study for efficacy (25).

## Opportunities for Antigen-Specific Treg Therapy in T1D

The finding that Treg induction potential is significantly limited during onset of islet autoimmunity (22) highlights the concept that antigen-specific Treg induction in the presence of ongoing autoimmune activation will benefit from combinatorial immune targeting. Specifically, a combination with treatments that control aberrant immune activation while fostering Tregs will be critical in order to broaden the window of opportunity for Treg induction.

### miRNA Targeting to Foster Tregs in Islet Autoimmunity

With the goal to understand mechanisms of impaired Treg induction, we focused on microRNAs (miRNAs). miRNAs are small non-coding RNAs that can sequence-specifically inhibit their target mRNAs. miRNAs usually target a multitude of different mRNAs, thereby regulating entire signaling pathways and complex cellular states, such as T cell activation, which makes them important targets for immunotherapies (26–28). Using miRNA sequencing of CD4^+^ T cells from peripheral blood of children with or without ongoing islet autoimmunity, we were able to identify several differentially regulated miRNAs and investigated three in more detail. Specifically, we focused on miRNAs that are predicted to target negative regulators of T cell activation and could therefore potentially inhibit Treg induction [reviewed in (29–31)].

We were able to demonstrate that miRNA92a-3p, a member of the miRNA17~92 cluster of miRNAs which was shown to induce lupus-like autoimmunity when overexpressed in mice (32), regulates human T follicular helper (TFH) cell differentiation (33). TFH cells are an integral part of the humoral immune response because of their ability to help B cells produce high-affinity antibodies [reviewed in (34)]. Accordingly, we found CXCR5^+^ insulin-specific TFH cell frequencies to be increased during onset of islet autoimmunity, which was directly correlated with miRNA92a-3p expression. Importantly, miRNA92a-3p targets negative regulators of T cell activation (e.g., PTEN, PHLPP2, FOXO1, CTLA4) and thereby simultaneously reduces Treg induction. Hence, inhibition of miRNA92a-3p enhanced while a miRNA92a-3p mimic reduced Treg induction (33).

Furthermore, we investigated miRNA181a-5p, which has been demonstrated previously to regulate the signal strength of the TCR stimulus in developing T cells in the thymus (35). In line with excessive T cell activation observed during recent onset of islet autoimmunity, we found miRNA181a-5p to be specifically increased in CD4^+^ T cells from peripheral blood of children with recent activation of islet autoimmunity. Importantly, we found that higher expression of miRNA181a-5p enhances the expression of Nfat5 involving mechanisms of increased TCR- and co-stimulation and that enhanced Nfat5 expression negatively affects Treg induction. Accordingly, inhibiting either miRNA181a-5p or Nfat5 augmented *in vitro* Treg induction, while inhibiting miRNA181a-5p in Nfat5 deficient T cells had no effect on Treg induction. These findings thereby highlight, that miRNA181a-5p mediated impairments in Treg induction are dependent on Nfat5 (22).

In a third study we used high throughput sequencing of RNA isolated by crosslinking immunoprecipitation (HITS-CLIP) to show, that miRNA142-3p directly targets the methylcytosine deoxygenase Tet2. Importantly, TET proteins catalyze the first step of DNA demethylation and can thereby impact the epigenetic landscape (36). We were able to link increased expression of miRNA142-3p and resulting reduced Tet2 expression with impairments both in Treg induction as well as in Treg stability. Accordingly, the inhibition of miRNA142-3p was able to enhance Treg induction and enable induced Tregs to retain their Foxp3 expression to a higher degree than their untreated counterparts (23).

Importantly, the inhibition of all three miRNAs or the downstream molecule Nfat5 directly *in vivo* in NOD mice with ongoing islet autoimmunity resulted in enhanced frequencies of Tregs accompanied by a reduction in the clinical disease score of the mice (22, 23, 33). These preliminary findings highlight the potential of miRNA-targeting as immunotherapy in T1D. Notably, a miRNA inhibitor is currently being investigated in a clinical trial as treatment for hepatitis C virus infections, thereby indicating the feasibility of miRNA modulation as immunotherapy (37). However, miRNAs are important regulators of cellular functions and can have distinct properties depending on the cell type. Therefore, the use of miRNA modulation as immunotherapy will be largely dependent on the cell type-specific targeting of the therapy. Specifically, the targeted delivery of miRNA inhibitors or mimics to immune cells or even immune cell subsets will greatly improve their use as immunotherapeutics. Here, it will be especially important to identify specific signatures for targeting defined subsets of immune cells, e.g., tissue-specific Tregs in the target organ, the pancreas.

### Targeting Tissue-Specific Tregs

Apart from their canonical function of immune suppression, it is now well accepted that Tregs likewise take residence in tissues, where they play important roles in maintaining tissue homeostasis. These tissue Tregs were found to express specific gene signatures that are distinct from their circulating counterparts. Such tissue specific Treg gene signatures have been identified for Tregs from specific tissues, while they have been especially well studied for Tregs in the muscle and adipose tissue [reviewed in (38)]. Importantly, some signature genes are universal for tissue Tregs while others are more unique to Tregs from distinct tissues, e.g., the expression of the transcription factor PPARγ on adipose-tissue residing Tregs (39). Apart from their gene expression signature, TCR sequencing of tissue resident Tregs has identified a distinct TCR repertoire and clonal expansion of certain TCRs, indicating the response to tissue-specific antigens (40). Importantly, treatment with the PPARγ agonist pioglitazone, which is used for the treatment of type 2 diabetes because of its positive effects on metabolic health and local inflammation, was shown to expand adipose tissue Tregs, which supports the idea of targeting tissue-specific Tregs for the treatment of diseases (39).

While Tregs in adipose tissue, muscle and the intestine have been studied extensively, only very little is known about Tregs in the pancreas. A study by the group of Christophe Benoist demonstrated that the diabetic lesions in NOD mice are enriched in CXCR3^+^ Tregs and that the expression of CXCR3 is dependent on Tbet. More importantly, they showed that the ablation of Tbet in Tregs accelerates the disease and overcomes the usually present sex-bias in NOD mice (41). Interestingly, Tbet^+^ Tregs were also found in the lamina propria of patients with inflammatory bowel disease (42) as well as in patients with multiple sclerosis (43), where Tbet^+^ Tregs were shown to contribute to the disease manifestation and being less suppressive (43). Importantly, the reduced suppressive activity was linked to the Ifnγ production of the Tregs which was not elevated in Tbet^+^ Tregs from the pancreas (41). These findings highlight the possibility of specifically targeting defined Treg subsets within the pancreas for a more tailored immune modulation. However, all studies conducted so far on pancreas residing Tregs focused solely on NOD mice with ongoing insulitis. A more detailed understanding of pancreas residing Tregs and their contribution to immune homeostasis in the steady state will be crucial to advance immune modulation targeted to the pancreas.

**Figure 1:**
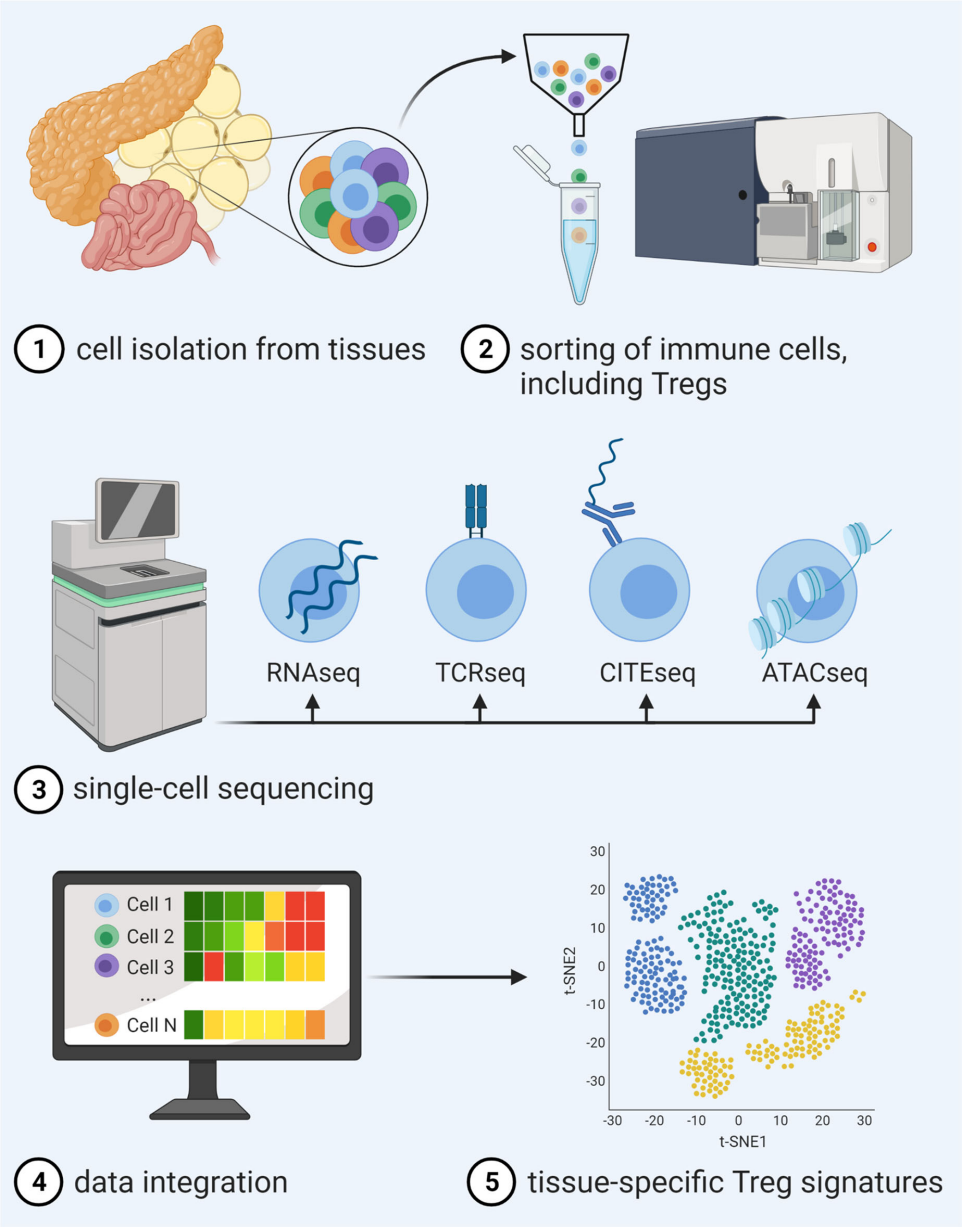
Advancements in single-cell multi-omics integration allow for a detailed analysis of tissue Treg signatures. After isolation and sorting of heterogeneous immune cell populations from tissues and single-cell sequencing of distinct libraries for RNAseq in combination with, e.g., TCRseq, CITEseq or ATACseq, novel computational approaches enable data integration of different traits, thereby enabling unprecedented description of molecular behavior and identities of individual cells within a certain tissue.

As one means to foster advancement in tissue-specific Treg targeting, recent years have seen tremendous progress in the simultaneous analysis of transcriptome, DNA methylation and accessibility, surface protein expression, perturbations, and receptor sequences on the single cell level. In this regard, computational strategies for integration of these complex data sets have enabled an unprecedented description of molecular behavior and identities of individual cells and therefore made it possible to move along to the next level of dissecting tissue Tregs (**Figure 1**).

### Defining Tissue-Specific Treg Characteristics Using Single-Cell Multi-Omics Integration

Current single-cell multi-omics methods can measure up to four different omics types at once [reviewed in (44–46)], with the transcriptomics layer often used to connect between the different omics types. These techniques bear high potential for medical research to study individual heterogeneity, drug resistance, or disease progression at an unprecedented level (47, 48). Especially, T cell focused immunological studies will benefit from recent developments as newly arising techniques can also simultaneously reconstruct TCR sequences and determine their specificities for a predefined set of epitopes (49–51). These methods have already greatly advanced our understanding of T cell responses in disease (50, 52–56), and lead to innovative analysis strategies such as the usage of TCR-sequence as natural barcodes to trace the cellular response pre- and post-antigen stimulation *in vivo* (57).

With the rise of single-cell multi-omics approaches, new computational models have been developed that can jointly analyze such multi-modal data [reviewed in (46, 58)]. Several studies used correlation-based approaches to jointly analyze copy number variations (59, 60), DNA methylation (61–63), or protein abundance (64) and gene expression data. Recently, Schattgen et al. proposed an integration approach for TCR and gene expression data based on graph analysis defined on transcriptomic and TCR distances and could uncover known and novel associations between TCR sequences and transcriptomics phenotypes (65). Others used traditional statistical approaches (66), or advanced deep learning methods (67–73) to integrate multiple data sources at once to represent the joint information of all omics-layers. Along these lines, a recent method by Zhang et al. jointly integrated TCR and transcriptomic information using Bayesian clustering based on the TCR sequence and gene expression profile (74). Through this method Zhang et al. could show that joint TCR and gene expression analysis better separates T-cell specificity and captures the antigen binding efficiency gradient better than TCR-information alone (74). Similarly, we introduced a joint TCR-transcriptome deep learning model which additionally captured transcriptional gradients within clonotypes (73). Such methods could be used to further elucidate the relationship between the TCR sequence and transcriptional information of Tregs in autoimmune diseases.

The identification of specific TCRs on tissue Tregs will help to define whether the migration of these cells to the tissue is likely antigen-driven and can also help to facilitate studies on tissue Tregs. In this regard, Diane Mathis group was able to analyze the ontogeny of visceral white adipose tissue (VAT)-residing Tregs by generating a mouse line transgenic for the TCR of an expanded VAT Treg clone (40). Additionally, the transfer of TCR transgenic Tregs has already been tested in preclinical studies for autoimmune diseases (75, 76). These studies mostly rely on the use of effector T cell derived TCRs and it is not entirely clear how that could affect Treg function, migration, and fate after transfer. The identification of tissue- and Treg-specific TCRs in the steady state as well as differences to the disease state might enable us to design such transgenic Tregs more strategically and could therefore help to increase efficacy and safety of TCR transgenic Treg infusions.

However, the identification of TCR sequences is only one side of the coin and a remaining bottleneck for T cell biology is the identification of the peptide-MHC ligands recognized by the identified TCRs. Here, recent advances have been made for experimental identification of epitopes recognized by orphan TCRs in a high-throughput screening of highly complex peptide-encoding oligo pools presented by bar-coded T cell-cytokine capturing APCs (77). Additionally, machine learning has enabled novel computational approaches to predict TCR specificity.

Sequence-based computational methods for TCR specificity analysis can be grouped into two categories: comparison and prediction. TCR comparison approaches impute antigen specificities by either allocating unknown TCRs to T-cell clusters or by assigning pairwise distance scores to TCR sequences with known antigen specificity. When several TCRs specific to the antigens of interest are known, these methods can be used to identify T cells with similar sequences likely to bind to the same antigen. The second category applies machine learning models to directly predict TCR binding to specific epitopes. Since these methods often additionally analyze the epitope sequence, they allow to predict specificity towards previously unknown antigens.

TCR sequences with common epitope specificity carry statistically enriched motifs (78, 79). Methods such as TCRdist (78) and GLIPH (79, 80) compare such common motifs to identify TCR sequences with shared antigen specificities. Other methods were proposed differing in computational approach to match TCRs using sequence similarity (81) or numeric embeddings (82, 83).

While comparison-based methods can serve as a proxy for determining TCR-specificity, such methods fail for novel epitopes without known corresponding TCRs. Machine learning methods can alleviate these issues by learning general rules that guide the T-cell epitope interaction. De Neuter et al. provided a proof of concept by predicting specificity towards one of two B*08 restricted HIV-1 epitopes based on the TCR CDR3β sequence (84). Jurtz et al. additionally incorporated the peptide sequence but observed limited generalization to unknown epitopes (85). Subsequently, different models developed on varying datasets have been proposed with limited improvements (86). In recent years, deep learning methods were introduced (52, 87–89), of which some incorporate additional information such as CDR3α, CDR1 and CDR2 sequences, HLA type, and surface protein counts leading partially to increased prediction performances (52, 86).

These tools will potentially enable the identification of Tregs associated with disease-relevant antigens by predicting the specificity for large libraries of sequenced T cells. By limiting the number of candidates, for which specificity needs to be tested, the time and cost for identifying disease-relevant Tregs will be significantly reduced. However, due to different evaluation methodologies and different datasets, these methods often cannot directly be compared. Therefore, it remains yet to be determined, which model to choose, and to what degree computational tools can be already used for the development of targeted immunotherapies. It is apparent though, that the use of multi-omics techniques for the deep characterization of tissue-specific Tregs can critically contribute to the development and advancement of Treg-based immunotherapies. TCR transgenic Tregs migrate to the site of immune activation and therefore will facilitate the development of effective and safe therapies. Additionally, identification of surface markers specific to tissue-residing Tregs will enable targeted delivery of therapeutics, e.g., miRNA inhibitors or mimics, to foster Tregs specifically at the site of the autoimmune attack.

## Conclusion

While advances have been made for antigen-specific Treg inducing therapies e.g. to treat patients with severe peanut allergies, the success of such therapies in autoimmune T1D is still limited. A broad impairment in Treg induction in children during onset of islet autoimmunity highlights the necessity of combinatorial strategies to foster Tregs in order to open the window of opportunity for antigen-specific Treg therapies. miRNA-targeting offers the opportunity to improve Treg induction and stability in T1D. However new strategies to specifically modify miRNAs in specific cell types are needed. Identifying key signatures and characteristics of Tregs residing in the pancreas, the target organ of the disease, will be important to target therapies more specifically to those cells that are directly involved in the disease development and progression. Major advances in the use of single-cell multi-omics integration together with machine learning approaches for TCR specificity prediction have paved the way for a detailed description of individual cells from different tissues and will therefore help to bring antigen-specific Treg therapy to the next level.

## Author Contributions

IS and FD reviewed the literature and wrote the manuscript. CD and BS reviewed the literature and contributed to the conceptualization of the manuscript. All authors contributed to the article and approved the submitted version.

## Funding

BS acknowledges financial support by the Postdoctoral Fellowship Program of the Helmholtz Zentrum München (https://www.helmholtz-muenchen.de/fellows/index.html). IS is supported by a Research Grant of the Deutsche Forschungsgemeinschaft (DFG, SE 3036/2-1). FD is supported by the Helmholtz Association under the joint research school “Munich School for Data Science - MUDS”. CD holds a professorship grant from the Excellence Program for Outstanding Female Scientists from the Helmholtz Association, is supported by a Research Group at Helmholtz Zentrum München, the German Center for Diabetes Research (DZD), through a membership in the CRC1054 of the Deutsche Forschungsgemeinschaft (B11), and through an award of the EFSD/JDRF/Lilly Programme on Type 1 Diabetes Research 2020.

## Conflict of Interest

The authors declare that the research was conducted in the absence of any commercial or financial relationships that could be construed as a potential conflict of interest.
